# Hypoglycemic Effect of Ethanol and Ethyl Acetate Extract of *Phellinus baumii* Fruiting Body in Streptozotocin-Induced Diabetic Mice

**DOI:** 10.1155/2015/783460

**Published:** 2015-06-28

**Authors:** Wen-Han Wang, Fei-Hua Wu, Yan Yang, Na Wu, Jing-Song Zhang, Na Feng, Chuan-Hong Tang

**Affiliations:** ^1^National Engineering Research Center of Edible Fungi, Key Laboratory of Applied Mycological Resources and Utilization, Ministry of Agriculture, Shanghai Key Laboratory of Agricultural Genetics and Breeding, Institute of Edible Fungi, Shanghai Academy of Agricultural Sciences, Shanghai 201403, China; ^2^Department of Pharmacology for Chinese Materia Medica, China Pharmaceutical University, 639 Longmian Avenue, Jiangning District, Nanjing, Jiangsu 211198, China

## Abstract

We investigated hypoglycemic effect of ethanol (EtOH) and ethyl acetate extract acetate (AcOEt) extracts in streptozotocin- (STZ-) induced diabetic mice. Our data showed the maximum inhibitory effect on the fasting plasma glucose (FPG) level was detected in STZ-induced diabetic mice administered with 400 mg/kg AcOEt extract of *P. baumii*. A lower glycated albumin (GA) level and a higher insulin level were observed in 400 mg/kg AcOEt and EtOH extract groups. Moreover, 400 mg/kg AcOEt and EtOH extract exhibited a stronger effect on increasing size and cell number of islets. The insulin expression level of *β*-cells and integrated optical density (IOD) value were significantly increased by the administration of 400 mg/kg AcOEt and EtOH extracts. Taken together, AcOEt and EtOH extracts of *P. baumii* fruiting body exhibited considerable hypoglycemic effect on STZ-induced diabetic mice.

## 1. Introduction

Increasing demand of natural products with antidiabetic activity is needed by patients with diabetes [1]. More than 30 traditional Chinese medicines are frequently used for the clinical treatment of diabetes and to treat associated complications. Medicinal fungi, such as* Tremella aurantia*,* Cordyceps sinensis,* and* Lentinus edodes,* are also natural medicines that possess antidiabetic activity [2–5].

*Phellinus baumii* as a species of medicinal mushrooms belongs to the Basidiomycotina, Hymenomycetes, Aphyllophorales, and Hymenochaetaceae, which is indigenous mainly in tropic America, Africa, and east Asia. It is shaped like a hoof, has a bitter taste, and in the wild grows on mulberry trees. The flesh is tough and woody, and its color ranges from dark brown to black. As one of medicinal mushrooms widely used in east Asia, especially Korea, China, and Japan,* P. baumii* has been used as a folk medicine in Asia. It is effective in improving blood circulation, suppressing cancer, enhancing body immunity, combating allergy and diabetes, curing oral ulcer, and alleviating gastroenteric disorders or lymphatic diseases [6–9]. Moreover,* P. baumii* also exhibits the antidiabetic effect [10–12]. The diabetic study revealed that oral administration of polysaccharides (EPS) has a hypoglycemic effect, resulting in reduced fasting plasma glucose (FPG) level in EPS-fed rats with enhanced glucose tolerance [11] and activated adipose tissue PPAR-*γ* and plasma PPAR-*γ* levels [12].* P. baumii* EPS can also stimulate the insulin secretion in diabetic rats [5].* P. baumii* consists of various bioactive substances, including polysaccharides, flavonoid, styrylpyrones, and phenolic compounds [6, 8]. Flavonoid, styrylpyrones, and phenolic compounds are mainly found in ethanol (EtOH) and ethyl acetate (AcOEt) extract of* P. baumii* fruiting bodies, and they have antioxidant and NF-*κ*B inhibitory activities [9]. Most of recent studies about the hypoglycemic effect of* P. baumii* have focused on polysaccharides derived from the water extract of fruiting body and mycelia, while very little attention has been paid to EtOH extract and other solvent extracts. Increasing evidence shows that oxidative stress is associated with the pathogenesis of diabetes. Oxidative stress causes a complex dysregulation of cell metabolism and cell-cell homeostasis. In particular, oxidative stress plays a key role in the pathogenesis of insulin resistance and *β*-cell dysfunction. In our previous work, we found these two extracts have better antioxidant activity in vitro model [13, 14].

In the present study, we prepared EtOH and AcOEt extracts from the fruiting bodies of* P. baumii* and evaluated their hypoglycemic effects. Our data provided a scientific basis for the application of* P. baumii* in treatments of diabetes.

## 2. Materials and Methods

### 2.1. Materials

Fruiting bodies of* P. baumii* were cultivated by Jin Zai Cultivating Professional Cooperatives of *Phellinus* in An Hui Province, China. Strains were obtained from the Herbarium of the Edible Fungi Culture Collection Center, Branch of China Culture Collection Center of Agricultural Microorganisms, and maintained at the Research Center of Medicinal Resources, IEF (accession number 3249). It also identified by ITS1–ITS4 sequencing data and matching with an identical sequence in GenBank.

### 2.2. Chemicals

Glucose assay kit (number 20110801) was purchased from Shanghai Rongsheng Biotechnology Co., Ltd. (Shanghai, China). Glycated albumin (GA) assay kit (number 20111001A) was provided by Nanjing Jiangcheng Bioengineering Institute (Nanjing, China). Insulin ELISA kit was obtained from R&D Systems Co., Ltd. (Minneapolis, MN). Streptozotocin (STZ, number S0130) was purchased from Sigma (St. Louis, MO, USA), and metformin hydrochloride tablets (number 090529) were supplied by Shanghai Sine Pharmaceutical Co., Ltd. (Shanghai, China).

### 2.3. Preparation of EtOH and AcOEt Extracts

Dried fruiting body of* P. baumii* was smashed in a blender, and the obtained crude powder was extracted with 80% EtOH for two times (reflux, 2 h of each time). Subsequently, collected solution containing extracts was concentrated in a rotary evaporator at 40°C, and the supernatant fluid was removed after it was stored at 4°C storage overnight. Finally, a solid EtOH extract was obtained through the freeze-drying method. For the AcOEt extract, the extract was dissolved in 20% EtOH and then sequentially partitioned with the same volume of petroleum ether, chloroform, AcOEt, and butanol for three times for 24 h. The AcOEt fraction was concentrated in a rotary evaporator under reduced pressure. Solid AcOEt extract was obtained by concentrating the AcOEt fraction using the freeze-drying method.

### 2.4. Animal Breeding Conditions and Treatments

This study was approved by the Animal Ethics Committee of China Pharmaceutical University. Male ICR mice (weight of 19–23 g and age of about 4 weeks) of clean grade, obtained from Comparative Medicine Center of Yangzhou University (License number SCXK (Su) 2007-0001), were housed in a controlled room under temperature of 24 ± 2°C and humidity of 55 ± 5% with a 12 h light/dark cycle. All mice were given free access to normal laboratory food and tap water. The care and treatment of these mice were performed in accordance with the Provisions and General Recommendation of Chinese Experimental Animals Administration Legislation.

After 1 week of acclimatization, 10 mice were randomly selected for the normal control group. Other mice were administered with STZ (160 mg/kg) via intravenous tail vein injection after a starvation of 15-16 h. Blood was collected from the ophthalmic venous plexus at 72 h after administration, and serum was separated for the FPG level detection. Mice with a FPG level of greater than 12 mmol/L were randomly divided into eight groups, including six sampling groups, one model control group, and one positive control group. In sampling groups, the diabetic mice were orally administered with AcOEt extract or EtOH extract at doses of 100, 200, and 400 mg/kg daily for 16 consecutive days. In the positive control group, mice were orally administered with metformin at a dose of 160 mg/kg daily for 16 days. Both normal control and model control groups were given 0.3% CMC-Na through oral administration for 16 days. The body weight was periodically monitored. On the 7th day, blood was collected from the ophthalmic venous plexus at 1.5 h after administration (after a starvation of 6 h) from all groups except for the normal control group, and then serum was separated for the FPG level detection. On 14th day, blood was collected again from the ophthalmic venous plexus at 1.5 h after administration (after a starvation of 6 h), and levels of FPG, GA, and insulin were determined. Moreover, hematoxylin-eosin (HE) and immunohistochemical staining was performed on pancreas tissues after the blood collection.

### 2.5. HE Staining of Pancreas Tissues

Pancreas tissues were postfixed in 10% formaldehyde solution, embedded in paraffin, and sectioned into slices of 4 *μ*m thickness. Subsequently, fixed tissue sections were stained with HE. Pathological changes of pancreatic islets were assessed using light microscope. Briefly, pathological changes mainly included the following contents: (1) the number of pancreatic islets per visual field (×100) was recorded; (2) the area of every islet per visual field was measured by image processing system (DP2-BSW) using Olympus microscope (Olympus BX51, Japan). Finally, the average area of each islet was calculated based on the islet number and islet area per field.

### 2.6. Insulin Expression in Pancreatic Islets by Immunohistochemical Staining

Pancreas was removed and postfixed in 4% paraformaldehyde for 4–6 h. Collected tissues were embedded in paraffin and sectioned into slices of 4 *μ*m thickness. Subsequently, formalin-fixed tissue sections were deparaffinized with xylene and rehydrated in a graded EtOH series. Sections were then incubated with 1 : 400 antibodies against insulin (number 3014) which was purchased from Cell Signaling Technology (Boston, MA, USA) at 4°C overnight. Staining intensity of all insulin-expressing cells in the entire area was evaluated using an image analysis system (Image-Pro Plus 5.0.1, Planetron, Tokyo) and then analyzed using the integrated absorbance method. The insulin expression was determined based on the integrated optical density (IOD).

### 2.7. Statistical Analysis

Results are presented as means ± standard deviation (SD). Intergroup comparisons were performed by one-way analysis of variance (ANOVA) and LSD's test. All of the variables were tested for normal and homogeneous variance by Levene's test. When necessary, Tamhane's* T*2 test was performed. A *P* value of less than 0.05 or 0.01 is significant and very significant, respectively.

## 3. Results 

### 3.1. Effect of* P. baumii* on the Body Weight Gain and FPG Level in STZ-Induced Diabetic Mice

Table 1 shows the effect of EtOH and AcOEt extracts from* P. baumii* on the body weight gain in STZ-induced diabetic mice. A significant difference in body weight gain was observed between 200 (*P* < 0.05) and 400 (*P* < 0.05) mg/kg AcOEt extract groups and the model control group at the 14th day.

Table 2 shows that the FPG level in model control group was significantly increased compared with the normal control group (*P* < 0.01). The FPG level of STZ-induced diabetic mice was significantly decreased by day 14 after administration of 200 and 400 mg/kg AcOEt extract and 400 mg/kg EtOH extract or 160 mg/kg metformin compared with the model control group (*P* < 0.05).

### 3.2. Effect of* P. baumii* on GA and Insulin Levels in STZ-Induced Diabetic Mice

At 14th day after administration, a significant GA-lowering effect was observed in diabetic mice treated with 200 and 400 mg/kg AcOEt extract or 160 mg/kg metformin (*P* < 0.05). In addition, such GA-lowering effect was also observed in diabetic mice treated with EtOH extract, showing 400 mg/kg EtOH extract significantly reduced GA level in diabetic mice (*P* < 0.05) (Table 3).

The serum insulin level of diabetic mice was markedly decreased (*P* < 0.05) compared with the normal control group. Similar effects on serum insulin level were observed using 400 mg/kg AcOEt extract compared with the positive control group (160 mg/kg metformin). In addition, our data also indicated that 400 mg/kg EtOH extract could induce a significantly (*P* < 0.05) increased serum insulin level (Table 3).

### 3.3. Effect of* P. baumii* on the Islet Morphology

Table 4 shows that the average islet number and islet area per field of model control group were significantly less than those of the normal control group (*P* < 0.01). We found that the average number of islets per field in groups administered with 200 and 400 mg/kg AcOEt was higher than that of the model control group (*P* < 0.05). The average number of islets could be also significantly increased after administration of 400 mg/kg EtOH extract, as well as 160 mg/kg metformin (*P* < 0.05).

Such effects were also observed on the average area of islets. The average islet area was increased by 400 mg/kg AcOEt, 200 and 400 mg/kg EtOH extract, and 160 mg/kg metformin (*P* < 0.05).

**Figure 1:**
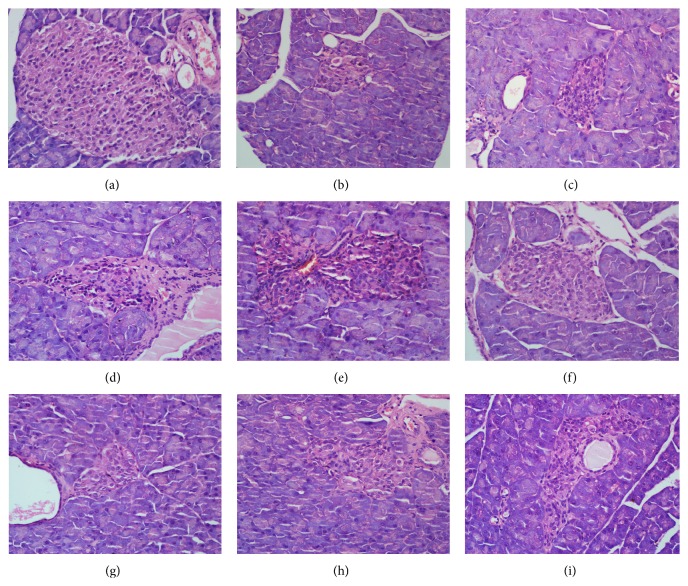
Pancreatic tissue slice from a normal control mouse (a) showed the normal tissue structure. The tissue slice from streptozotocin-induced diabetic mouse (b) showed abnormal morphology of islets. The tissue sections from type 2 diabetic mice that received the AcOEt extract (100 mg/kg, 200 mg/kg, and 400 mg/kg; (c), (d), and (e), resp.) or EtOH extract (100 mg/kg, 200 mg/kg, and 400 mg/kg; (g), (h), and (i), resp.) exhibited the increased size and cell number of islets. Original magnification 100x and H&E staining.

Pathological examination showed that large-volumed and round rope-shaped islets with regular and clear edge were observed in the normal control group. A large number of *β*-cells were evenly distributed in islets and rich cytoplasm. Light staining and round nucleus were also observed in islets of the normal control group. In contrast, small-volumed and irregularly shaped islets were observed in model control group, with a reduced number of *β*-cells, and disarranged and vacuolated degeneration of cells. Interestingly, the size and cell number of islets were increased after administration of 100, 200, and 400 mg/kg AcOEt extract, 200 and 400 mg/kg EtOH extract, or 160 mg/kg metformin, which led to formation of clear islet edge and orderly arranged cells (Figure 1).

### 3.4. Effect of* P. baumii* on the Insulin Expression

**Figure 2:**
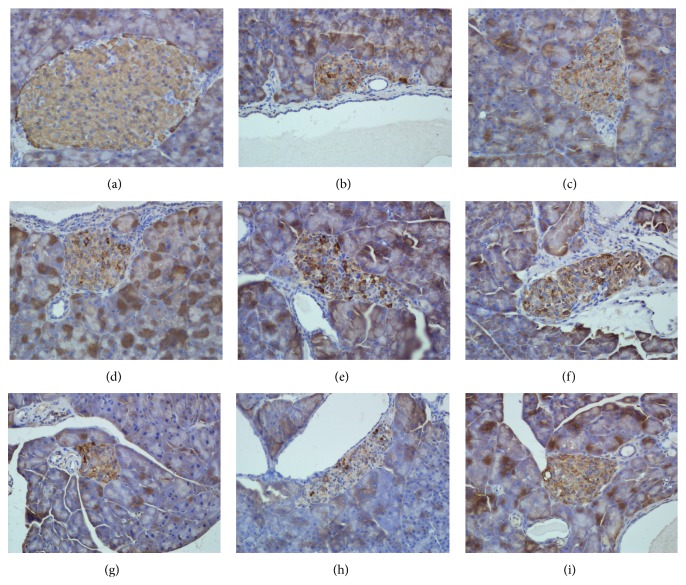
Effect of* P. baumii* on insulin-expressing *β*-cells. (a) Control group. (b) Streptozotocin-induced diabetic mouse group. (c), (d), and (e) The groups were treated with 100 mg/kg, 200 mg/kg, and 400 mg/kg AcOEt extract, respectively. (g), (h), and (i) The groups were treated with 100 mg/kg, 200 mg/kg, and 400 mg/kg EtOH extract, respectively. Original magnification 100x and immunohistochemical staining.

Table 5 and Figure 2 demonstrated that *β*-cells were high-density, orderly arranged, and equally distributed in islets of the normal control group. We found that the number of insulin-expressing *β*-cells in model control groups was lower than that of the normal control group. Moreover, a significantly reduced IOD value in model control group was observed in the insulin-expressing area (*P* < 0.01). The insulin expression of *β*-cells and IOD value of insulin-expressing area were significantly increased in the 400 mg/kg AcOEt group compared with the model control group (*P* < 0.05). In addition, an increased effect on the insulin expression was detected in 400 mg/kg EtOH extract or 160 mg/kg metformin groups (*P* < 0.05).

## 4. Discussion

Diabetes mellitus is a chronic disease that results from the body's inability to sufficiently produce and/or properly use insulin. More than 347 million people worldwide have diabetes according to the report of the World Health Organization [15]. By 2030, more than 366 million people globally will have diabetes. Medicinal mushrooms have commonly been used as therapeutic agents for various diseases in Asia, which are widely prescribed even when their biologically active compounds are unknown due to their effectiveness, limited side effect, and relatively low cost [16]. In recent years, Nature products have been extensively investigated due to its capacity to combat diabetes [17, 18], making* P. baumii*, which belongs to nature products, a prospective candidate for developing novel antidiabetic compounds from natural resources.

In our study, we investigated the hypoglycemic effect of AcOEt and EtOH extracts from* P. baumii* using a STZ-induced diabetic mice model that closely recapitulated the pathophysiology with mild type I diabetes. As a broad-spectrum antibiotic extracted from Streptomyces achromogenes, STZ (N-nitroso derivative of glucosamine) is particularly toxic to insulin-producing *β*-cells of the pancreas and widely used to induce insulin-dependent diabetes mellitus in experimental animal models. Most drugs for diabetic therapy have focused on blood glucose control [16]. Insulin is a hormone that regulates the glucose level. Type I diabetes, known as insulin-dependent diabetes, is a chronic condition whereby the pancreas produces little or no insulin. Therefore, levels of GA and insulin were selected as the key indexes of diabetes in this study. We found that AcOEt and EtOH extracts induced a decreased GA level by regulating insulin secretion since the GA level (free glucose level) is controlled by insulin. Oxidative stress with glucotoxicity and lipotoxicity is diabetes-related phenomena, which is involved in the pathogenesis of *β*-cell dysfunction [19, 20]. The STZ treatment inhibits the insulin secretion through oxidant destruction of *β*-cells in the pancreatic islets. In this study, we found that inoscavin A and flavonoid were enriched in AcOEt and EtOH extracts, and we showed that these extracts demonstrated antioxidant activity in our studies [13, 14].

Therefore, we assumed that AcOEt and EtOH extracts from* P. baumii* played a critical role in repairing *β*-cells destruction by inhibiting the free radical scavenging activity and then promoting insulin synthesis. To test this, we evaluated islet morphology and insulin secretion of *β*-cells. Our data indicated the AcOEt extract could maintain the islet morphology and promote the insulin production. This finding was consistent with previous study showing similar effect on the protection of *β*-cells using the extract from* P. baumii* [21].

In the present study, we focused on the hypoglycemic effect of small molecule substances from fruiting body, whereas others reported the hypoglycemic effect of mycelium polysaccharides. We found that the compositions of fruiting body were different from those of mycelium, and other studies also reported the significant difference in terms of compositions between fruiting body and mycelium from* Ganoderma tsugae* [22].

Taken together,* P. baumii* administration had beneficial effects on increased blood glucose level induced by STZ, leading to a significantly improved diabetic situation. Moreover, our data also showed that* P. baumii* had potential to prevent diabetes. In addition, since molecular signaling pathway of* P. baumii* hypoglycemic effect is unclear, further investigation is required to clarify this mechanism.

## Figures and Tables

**Table 1:** Effect of *P. baumii* on the body weight gain of diabetes mice.

Group	Dose (mg/kg)	Animal number^a^ (piece)	First day	Fifth day	Seventh day^b^	Ninth day^c^	Thirteenth day	Fourteenth day
Control	—	10	21.1 ± 1.7	26.2 ± 1.2	28.8 ± 1.6	30.7 ± 2.1	33.7 ± 2.3	34.9 ± 2.4

Model	—	9	20.3 ± 1.5	20.2 ± 1.5^##^	19.6 ± 1.7^##^	19.7 ± 1.4^##^	19.0 ± 1.9^##^	18.8 ± 1.6^##^

AcOEt extract	100	9	20.3 ± 1.6	20.1 ± 1.9	20.3 ± 2.3	20.1 ± 2.2	20.3 ± 2.6	21.1 ± 2.8
	200	10	20.5 ± 1.7	20.8 ± 1.6	20.6 ± 2.4	20.1 ± 2.1	21.1 ± 2.3	22.2 ± 2.0^*∗*^
	400	9	20.4 ± 1.4	19.9 ± 1.7	20.3 ± 2.5	20.2 ± 2.1	20.2 ± 2.1	21.3 ± 2.2^*∗*^

EtOH extract	100	10	20.2 ± 2.0	20.1 ± 2.5	19.2 ± 2.4	19.6 ± 2.4	19.5 ± 1.8	19.9 ± 2.2
	200	9	20.4 ± 1.5	20.4 ± 2.1	20.3 ± 2.0	20.0 ± 1.9	19.7 ± 2.1	20.2 ± 2.3
	400	10	20.3 ± 1.9	20.4 ± 2.3	20.0 ± 2.2	19.9 ± 1.5	20.3 ± 2.8	20.6 ± 2.2

Metformin	160	10	20.4 ± 1.7	20.3 ± 2.1	20.8 ± 1.6	20.7 ± 1.6	20.2 ± 1.2	22.1 ± 1.5^*∗*^

^##^ *P* < 0.01 versus normal control group; ^*∗*^ *P* < 0.05, ^*∗∗*^ *P* < 0.01 versus model control group.

^a^Mice number of each group at fourteenth day.

^b^Mice number of each group was ten at seventh day.

^c^Mice numbers of 100 mg/kg AcOEt extract group and mode group were nine, and number of the other group was ten at ninth day.

**Table 2:** Effect of *P. baumii* on the FPG level of diabetic mice.

Group	Dose (mg/kg)	Animal number^a^ (piece)	FPG (m mol/L)	
			7th day^Ф^	14th day
Control	—	10	6.75 ± 0.86	6.37 ± 0.73

Model	—	9	22.18 ± 6.06^#^	20.45 ± 4.91^##^

AcOEt extract	100	9	19.70 ± 4.98	15.41 ± 5.09
	200	10	17.04 ± 3.90	12.88 ± 4.93^*∗*^
	400	9	15.87 ± 6.76	11.71 ± 4.59^*∗*^

EtOH extract	100	10	22.82 ± 6.57	17.06 ± 5.54
	200	9	18.65 ± 7.76	14.36 ± 4.63
	400	10	16.51 ± 5.72	13.30 ± 5.03^*∗*^

Metformin	160	10	15.45 ± 7.53^*∗*^	8.27 ± 2.85^*∗*^

^#^ *P* < 0.05, ^##^ *P* < 0.01 versus normal control group; ^*∗*^ *P* < 0.05, ^*∗∗*^ *P* < 0.01 versus model control group.

^a^Animal number of each group at 14th day.

^Ф^Sample number of each group is 10 at 7th day.

**Table 3:** Effect of* P.  baumii* on GA and serum insulin levels of diabetic mice induced by STZ.

Group	Dose (mg/kg)	Animal number^a^ (piece)	GA (m mol/L)	Insulin (mIU/L)
Control	—	10	1.66 ± 0.10	13.15 ± 1.97

Model	—	9	2.08 ± 0.23^#^	7.74 ± 1.32^#^

AcOEt extract	100	9	1.82 ± 0.27	9.35 ± 1.34
	200	10	1.68 ± 0.30^*∗*^	9.75 ± 1.17
	400	9	1.70 ± 0.25^*∗*^	10.98 ± 1.61^*∗*^

EtOH extract	100	10	1.89 ± 0.18	9.03 ± 1.65
	200	9	1.77 ± 0.27	9.42 ± 1.24
	400	10	1.74 ± 0.24^*∗*^	10.02 ± 1.48^*∗*^

Metformin	160	10	1.85 ± 0.24^*∗*^	10.17 ± 1.99^*∗*^

^#^ *P* < 0.05,^##^ *P* < 0.01 versus normal control group; ^*∗*^ *P* < 0.05, ^*∗∗*^ *P* < 0.01 versus model control group.

^a^Animal number of each group at 14th day.

**Table 4:** Effect of *P.  baumii* on morphology of islet.

Group	Dose (mg/kg)	Animal number^a^ (piece)	Islet number (piece/pcs)	Islet area (*µ*m^2^/pcs)
Control	—	10	1.25 ± 0.27	8561 ± 1734

Model	—	9	0.13 ± 0.07^##^	1633 ± 359^##^

AcOEt extract	100	9	0.31 ± 0.18	2162 ± 707
	200	10	0.43 ± 0.25^*∗*^	2349 ± 1057
	400	9	0.41 ± 0.22^*∗*^	2989 ± 1231^*∗*^

EtOH extract	100	10	0.23 ± 0.16	2025 ± 1235
	200	9	0.30 ± 0.18	2643 ± 1511^*∗*^
	400	10	0.33 ± 0.18^*∗*^	2781 ± 1548^*∗*^

Metformin	160	10	0.35 ± 0.21^*∗*^	2593 ± 1234^*∗*^

^##^ *P* < 0.01 versus normal control group; ^*∗*^ *P* < 0.05, ^*∗∗*^ *P* < 0.01 versus model control group.

^a^Mice number of each group at 14th day.

**Table 5:** Effect of *P.  baumii* on insulin expression.

Group	Dose (mg/kg)	Animal number^a^ (piece)	Insulin expression level (×10^3^)
Control	—	10	162.5 ± 35.3

Model	—	9	21.3 ± 8.2^##^

AcOEt extract	100	9	34.0 ± 11.9
	200	10	46.3 ± 11.9
	400	9	83.0 ± 31.1^*∗*^

EtOH extract	100	10	23.6 ± 14.0
	200	9	35.5 ± 18.5
	400	10	44.1 ± 12.8^*∗*^

Metformin	160	10	55.6 ± 19.5^*∗*^

^##^ *P* < 0.01 versus normal control group; ^*∗*^ *P* < 0.05, ^*∗∗*^ *P* < 0.01 versus model control group.

^a^Mice number of each group at 14th day.
